# Seminal plasma exosomes improve the motility and mitochondrial function of goat spermatozoa during liquid storage by regulating oxidative phosphorylation

**DOI:** 10.1007/s44154-025-00253-6

**Published:** 2025-10-28

**Authors:** Tengfei Liu, Mengmei Zhang, Xinkang Li, Xinyan Zhao, Yongjie Wu, Hong Chen

**Affiliations:** 1 College of Veterinary Medicine, Northwest A&F University, Yangling, Shaanxi Province 712100 China; 2 Department of Radiology, Hospital of Northwest A&F University, Yangling, Shaanxi Province 712100 China

**Keywords:** Goat spermatozoa, Seminal plasma exosomes, Liquid storage, Motility, Oxidative phosphorylation

## Abstract

Exosomes as bilayer membranous vesicles are abundant in seminal plasma and mediate intercellular communication by transferring active biomolecules. Numerous studies have revealed the involvement of exosomes in regulating various biological properties of spermatozoa. However, the beneficial roles of seminal plasma exosomes in maintaining spermatozoon motility and mitochondrial function during liquid storage have not yet been unexplored in goat. In this study, the reduction of ATP content in goat spermatozoa was detected along with the decrease in spermatozoon motility under liquid storage, and the level of oxidative phosphorylation was also decreased. The interaction of exosomes and spermatozoon mitochondria was observed using high pressure freezing/freeze-substitution in combination with transmission electron microscope. Seminal plasma exosomes of goat were isolated and used to incubate with spermatozoa, and the binding and fusing of exosomes with spermatozoa was further validated. Furthermore, the addition of seminal plasma exosomes exhibited an increase in motility and oxidative phosphorylation in liquid-stored spermatozoa. Several mitochondrial functional parameters, including mitochondrial membrane potential, the levels of mitochondrial ROS and intracellular Ca^2+^, and the copy number and integrity of mitochondrial DNA, were also improved in spermatozoa after incubating with exosomes. Notably, the level of TFAM protein was increased in exosome-treated spermatozoa, indicating that the enhanced proteins may be delivered by exosomes to spermatozoa. These results suggest that seminal plasma exosomes could improve spermatozoon motility and mitochondrial function by regulating oxidative phosphorylation, which would provide insights into the understanding of protective roles of exosomes in goat spermatozoa during liquid storage.

**Supplementary Information:**

The online version contains supplementary material available at 10.1007/s44154-025-00253-6.

## Introduction

Liquid storage is one of the important semen storage technologies that greatly facilitate the development of artificial insemination and goat breeding industry (Faigl et al. 2012; Zuidema et al. 2021). Currently, although many efficient methods, such as the addition of different extenders, have been applied for prolonging the storage time of spermatozoa, the major challenge to the improvement of liquid storage technology is still to control the decline in the motility of spermatozoa (Yang et al. 2018; Ren et al. 2019; Liu et al. 2019). Enough adenosine triphosphate (ATP) to sustain the availability of energy is required for the motility of spermatozoa, as well as the consequent fertilization capacity (Moraes and Meyers 2018; Ferramosca and Zara 2014). Mitochondria are recognized as essential for spermatozoon motility and play principal roles in cellular metabolism and energy production through ATP synthesis (Moraes and Meyers 2018; Boguenet et al. 2021). Spermatozoa possessing an extremely particular mitochondrial organization are typically more prone to suffer the structural and functional damage of mitochondria under liquid storage, causing the reduction in energy production (Boguenet et al. 2021; Durairajanayagam et al. 2021).

The ATP generation in mammalian spermatozoa is demonstrated to utilize two metabolic pathways: glycolysis occurring in the flagellum of principal piece, and oxidative phosphorylation (OXPHOS) taking place in the mitochondria of mid-piece (Ferramosca and Zara 2014; Tourmente et al. 2015). Mitochondrial respiration coupled to OXPHOS providing the main energy and ATP production for the motility of spermatozoa has been reported in various species (Varner et al. 2015; Davila et al. 2016; Papa et al. 2018). The mitochondrial OXPHOS system involves the coordinated activity of the mitochondrial electron transport chain (ETC) and consists of five multi-enzymatic complexes, from I to V (Boguenet et al. 2021; Gualtieri et al. 2021). Numerous evidences highlight that the impairments of mitochondrial ETC, such as the enzymatic activity of the respiratory chain complexes and mitochondrial respiration efficiency, are directly related to the defects in the spermatozoon quality parameters (Boguenet et al. 2021; Ferramosca et al. 2012). Mitochondrial DNA (mtDNA) encodes 13 protein subunits of ETC complexes that include the crucial components of the mitochondrial OXPHOS pathway, and the functional mtDNA determines the mitochondrial function (Boguenet et al. 2021; Popova et al. 2022). Studies on male infertility have found that the decreased spermatozoon quality is associated with the alteration of mtDNA copy number, the reduction in mtDNA integrity, and the aberrant expression of mtDNA regulators (Gualtieri et al. 2021; Song and Lewi 2008; Rosati et al. 2020). The maintenance of mtDNA stability is primarily mediated by mitochondrial transcription factor A (TFAM), which is a nuclear-encoded transcription factor and plays critical roles in mtDNA replication and transcription (Rantanen et al. 2001; Song et al. 2020). Moreover, the downregulation of mitochondrial TFAM protein level is correlated with the mtDNA damage and mitochondrial dysfunction (Rantanen et al. 2001; Zhao et al. 2021; Gao et al. 2022).

Seminal plasma accounting for 95% of semen contains important excellent components and abundant extracellular vesicles that seem to play critical roles in spermatozoon in vitro preservation (Höfner et al. 2020). Exosomes, one of the extracellular vesicles with double membrane structure ranging in size from 30 to 150 nm, are extensively found in seminal plasma (Guo et al. 2023; He et al. 2018). Increasing reports have indicated that seminal plasma exosomes (spEXs) may mediate intercellular communication by transferring a large variety of biological components and bioactive cargo (such as protein and RNA) to spermatozoa, which modifies the biological properties and influences the biological functions in spermatozoa (He et al. 2018; Vilanova-Perez et al. 2020). Studies have suggested that the addition of spEXs could improve mitochondrial membrane potential (MMP), plasma membrane integrity, motility, capacity, and structure of spermatozoa during in vitro preservation (Mahdavinezhad et al. 2022; Wang et al. 2022; Kowalczyk and Kordan 2024; Parra et al. 2024). However, the potential mechanisms and protective roles of spEXs in regulating the liquid storage of goat spermatozoa is still unclear. Therefore, in this study, the major aims were to investigate the effects of exosomes from goat seminal plasma on the quality of goat spermatozoa and elucidate the vital interaction relationship between spEXs and spermatozoa. The morphological evidence for the binding and fusing of spEXs with liquid-stored spermatozoa was first provided. Moreover, the positive roles of spEXs as effective supplement in alleviating the liquid storage-induced mitochondrial damage to goat spermatozoa was validated. These findings could provide a solid foundation for the better understanding of exosome application and facilitate the improvement of liquid storage technology of goat spermatozoa.

## Results

### Liquid storage decreased the motility and energy production of goat spermatozoa

**Fig. 1:**
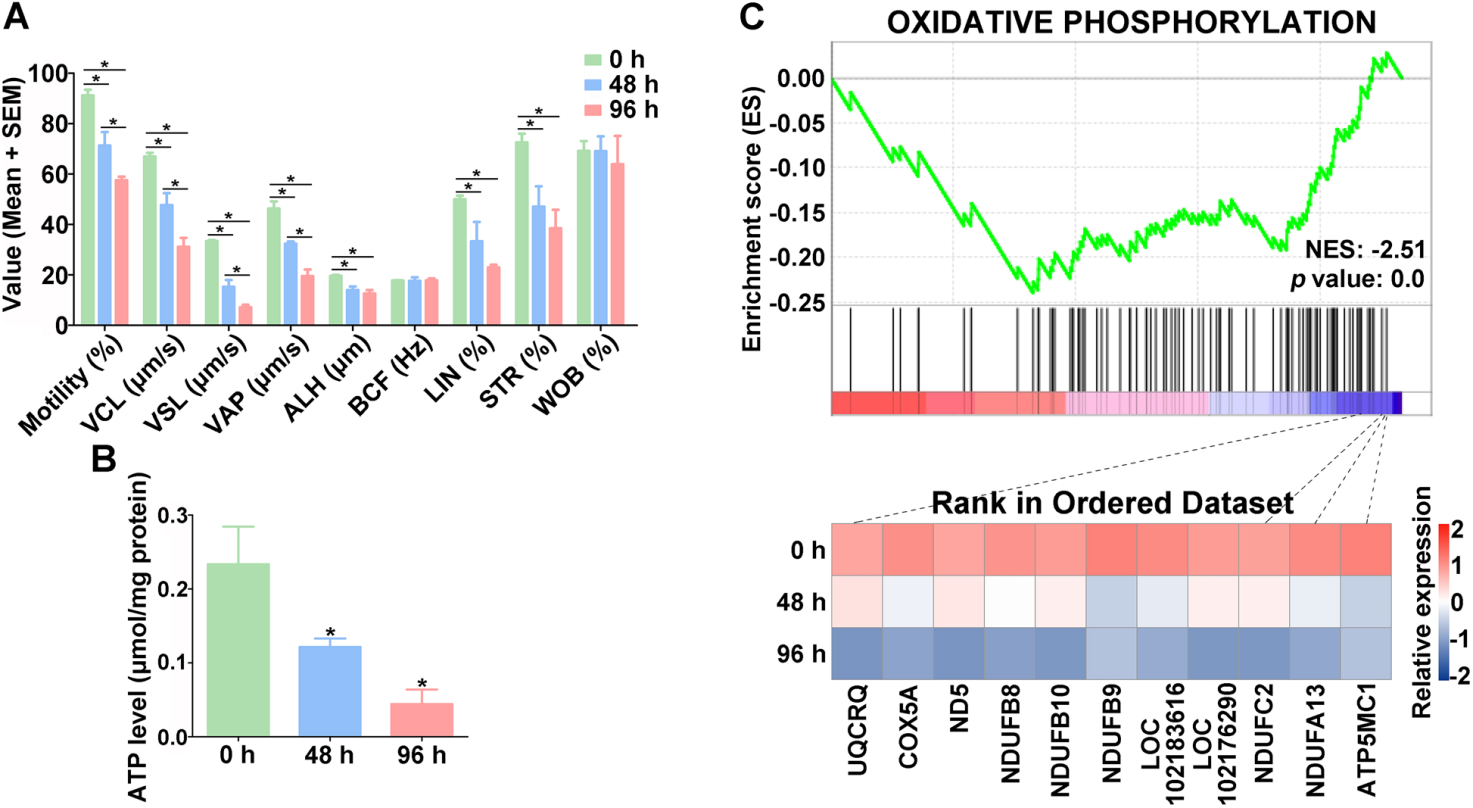
Assessment of motility parameters (**A**) and ATP level (**B**) and enrichment analysis of oxidative phosphorylation pathway (**C**) in goat spermatozoa under liquid storage. VCL, curvilinear velocity. VSL, straight-line velocity. VAP, average path velocity. ALH, amplitude of lateral head displacement. BCF, beat cross frequency. LIN, linearity. STR, straightness. WOB, wobble. *P < 0.05

Analysis of goat spermatozoon motility showed that the motility of spermatozoa was significantly reduced (*p* < 0.05) both at 48 h and 96 h under 4 °C liquid storage (Fig. 1A). The motility of liquid-stored spermatozoa was more than 90% at 0 h, while it was reduced to 71.35% at 48 h and 57.58% at 96 h. Further analysis showed that curvilinear velocity (VCL), straight-line velocity (VSL), average path velocity (VAP), amplitude of lateral head displacement (ALH), linearity (LIN), and straightness (STR) of goat spermatozoa were also significantly reduced (*p* < 0.05) both at 48 h and 96 h during liquid storage (Fig. 1A). There was no significant difference in beat cross frequency (BCF) and wobble (WOB) of spermatozoa during liquid storage. Moreover, changes in energy production of goat spermatozoa after liquid storage were further evaluated. The results showed that the level of ATP was significantly decreased (*p* < 0.05) at 48 h and 96 h in liquid-stored spermatozoa (Fig. 1B).

### Liquid storage decreased the OXPHOS level of goat spermatozoa

To investigate the effects of liquid storage on energy metabolism related pathways in spermatozoa, the enrichment analyses of glycolysis/gluconeogenesis and OXPHOS pathways were performed based on our previous data of TMT-based quantitative proteomic analysis (Liu et al. 2019). Gene set enrichment analysis (GSEA) showed that there was no significant difference in the glycolysis/gluconeogenesis pathway during liquid storage, while the pathway of OXPHOS exhibited significant enrichment (*p* < 0.01) in the comparison groups of 96 h/0 h and 96 h/48 h (Fig. 1C; Fig. S1). Meanwhile, 11 proteins involved in OXPHOS pathway, such as UQCRQ, COX5A, and ND5, exhibited down-regulated expression after liquid storage (Fig. 1C).

**Fig. 2:**
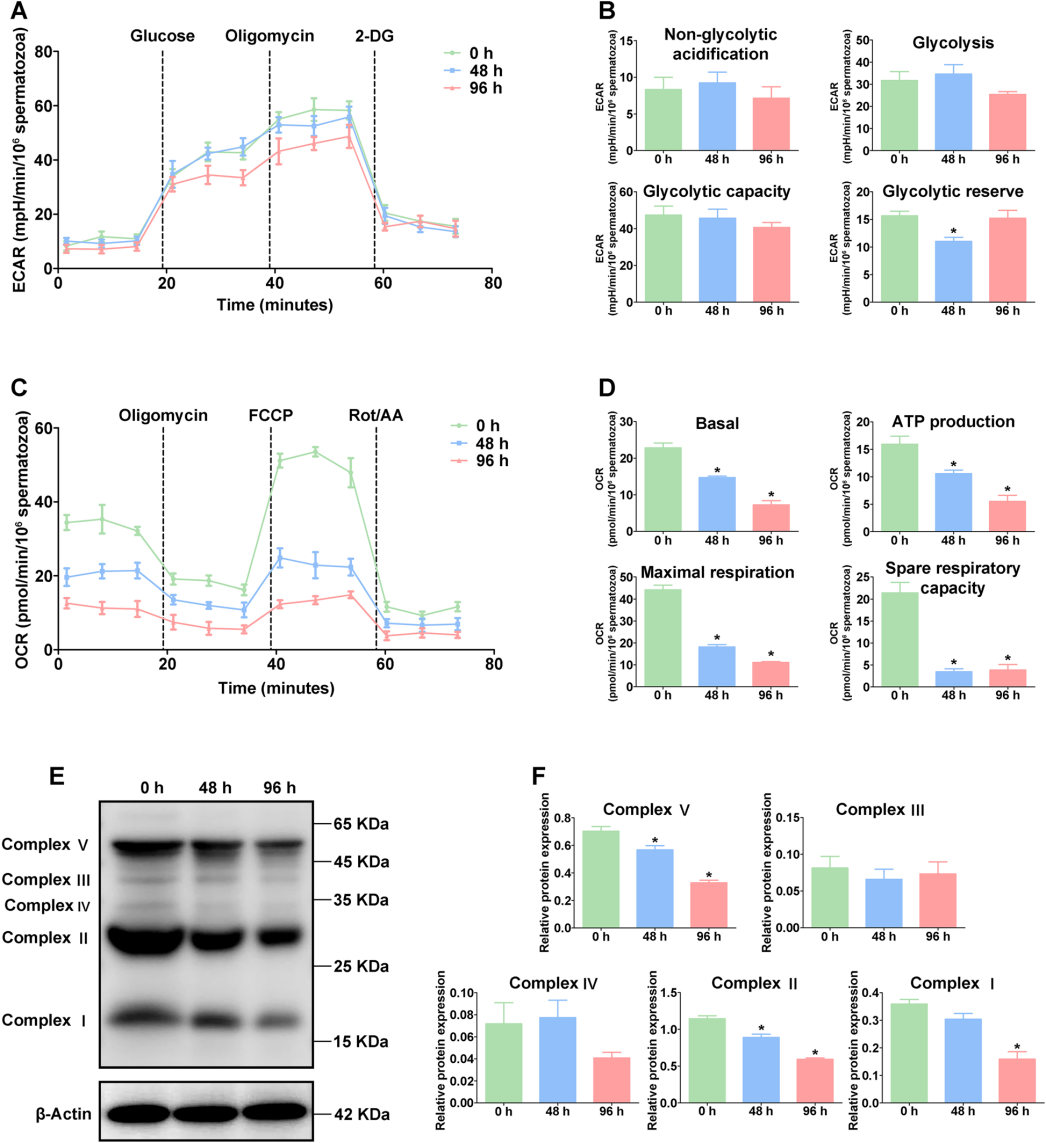
Measurement of extracellular acidification rate (**A**, **B**) and oxygen consumption rate (**C**, **D**) and Western blot analysis of oxidative phosphorylation complex protein expression (**E**, **F**) in goat spermatozoa under liquid storage. ECAR, extracellular acidification rate. OCR, oxygen consumption rate. *P < 0.05 compared to 0 h

Moreover, the extracellular acidification rate (ECAR) assay revealed that there was no significant difference in ECAR levels under liquid storage, including non-glycolytic acidification, glycolysis, and glycolytic capacity (Fig. 2A, B). Remarkably, the oxygen consumption rate (OCR) analysis showed a significant decrease (*p* < 0.05) in basal respiratory, ATP production, maximal respiratory, and spare respiratory capacity of goat spermatozoa at 48 h and 96 h after liquid storage (Fig. 2C, D). Furthermore, Western blot analysis showed that the expression levels of OXPHOS complex V, II, and I were significantly decreased (*p* < 0.05) at 96 h after liquid storage (Fig. 2E, F). These results indicated that liquid storage induced the reduction in OXPHOS of goat spermatozoa.

### Ultrastructural observation of interaction between goat spermatozoa and exosomes during liquid storage

**Fig. 3:**
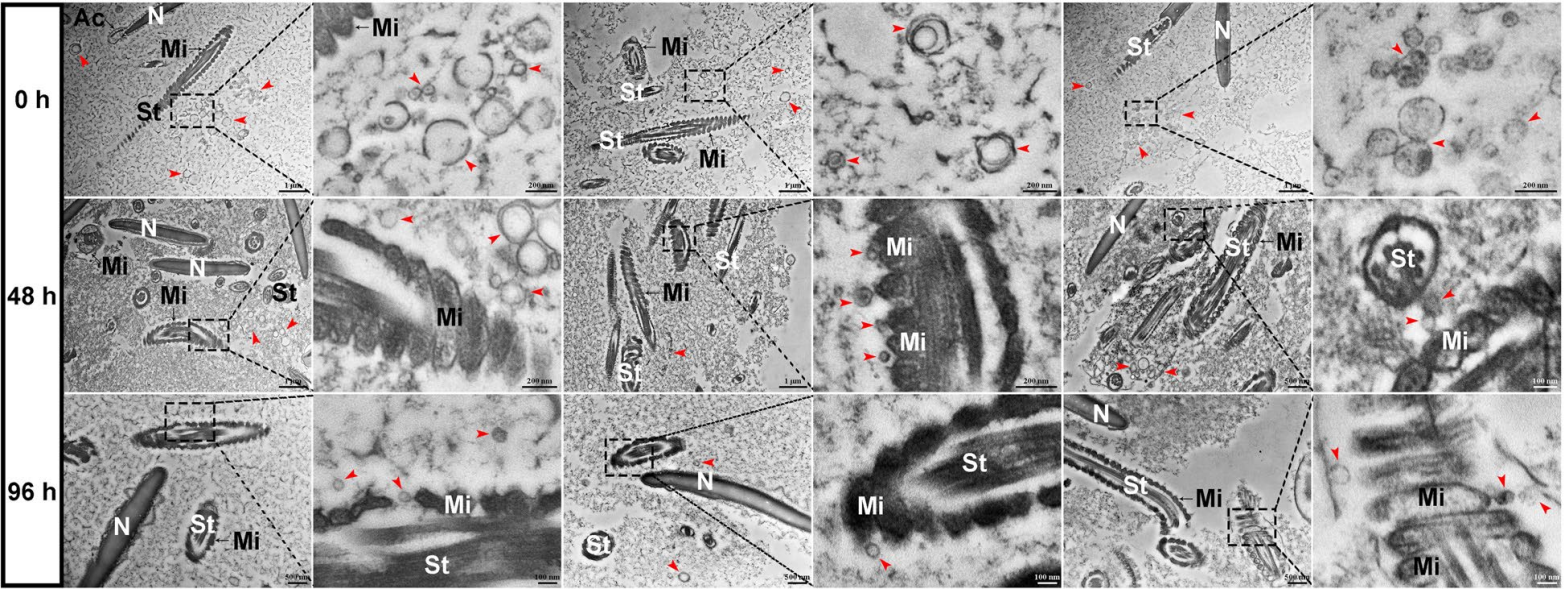
Images by high pressure freezing/freeze-substitution in combination with transmission electron microscopy showing morphologic changes in goat spermatozoa and the binding and capture process of exosomes to goat spermatozoa during liquid storage. Mi, mitochondria. St, spermatozoa tail. Ac, acrosome. N, Nuclear. The red arrows indicate the double-membrane exosomes

The technique of high pressure freezing/freeze-substitution (HPF/FS) possessing the advantage of more realistically displaying cellular ultrastructure has been widely used for providing samples with superior ultrastructural preservation (Jadav et al. 2023; Sosinsky et al. 2008; Velamoor et al. 2020). In this study, the HPF/FS in combination with transmission electron microscope (TEM) were performed to observe the ultrastructural changes of semen under liquid storage (Fig. 3). The normal structural characteristics in spermatozoa were observed at 0 h (the fresh semen), while the spermatozoa at 48 h and 96 h after liquid storage exhibited morphological abnormality, such as plasma membrane swelling, acrosome damage, and mitochondrial swelling and deformity. Notably, a larger number of bilayer membranous small vesicles with a diameter of 50 to 150 nm, which represented the typical characteristics of spEXs (marked by red arrows), were found in the semen and surrounded the spermatozoa (Fig. 3). More importantly, it was appeared that some spEXs adhered to and fused with the spermatozoon mitochondria at 48 h and 96 h. These results suggested that spEXs may play potential roles in fusing and interacting with spermatozoon mitochondria during liquid storage.

### Validation of spEXs interacting with goat spermatozoa

**Fig. 4:**
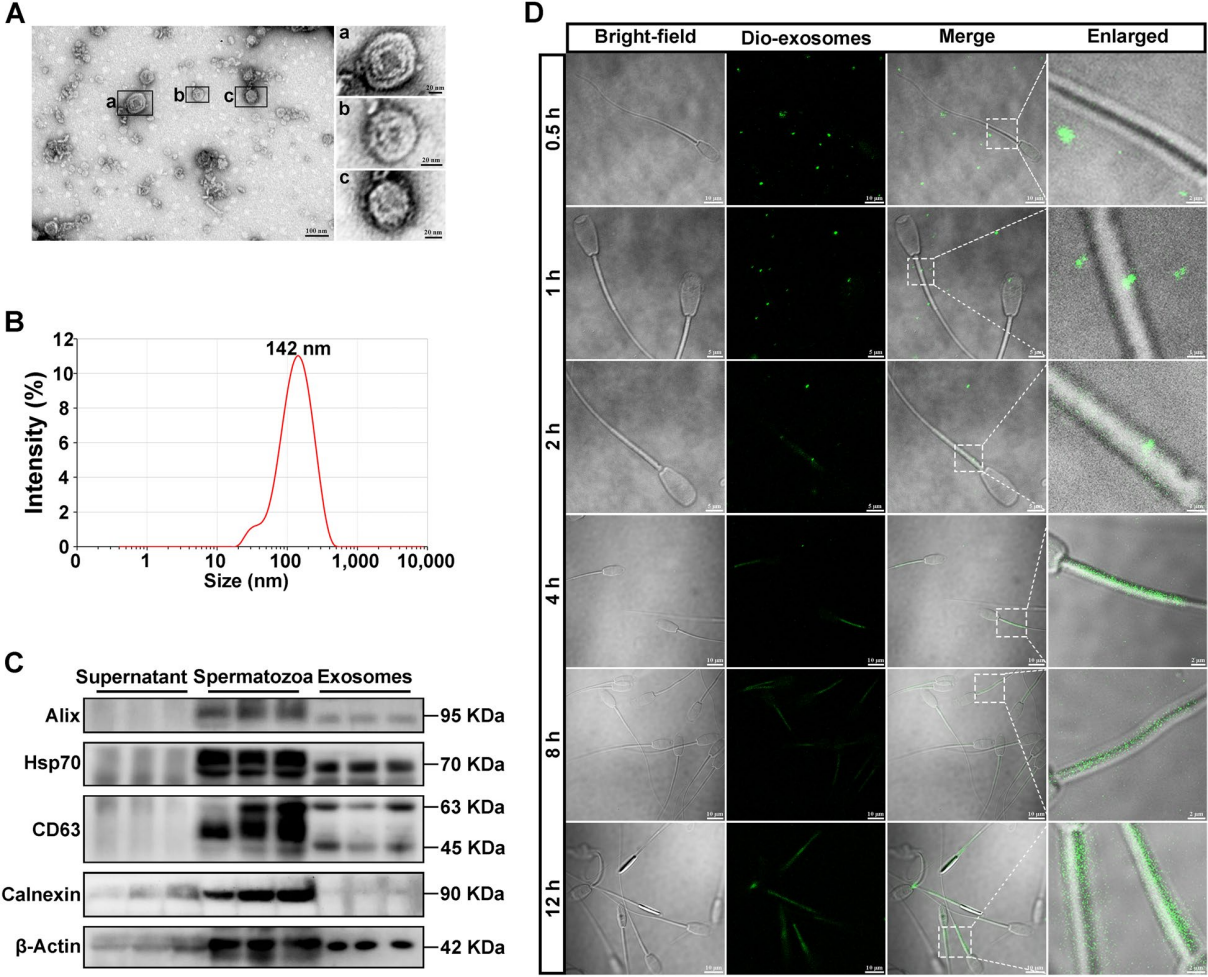
Validation of exosome extraction from goat seminal plasma and the fusing of exosome with goat spermatozoa. **A** Observation of exosome morphology. **B** Detection of exosome size distribution. **C** Expression of Calnexin protein and exosome marker protein Alix, HSP70 and CD63 by Western blot analysis. Calnexin protein is used as a negative control. **D** Observation of the fusing of exosomes with goat spermatozoa. The spermatozoa were incubated with DiO-labeled exosomes that was represented by green fluorescence

The exosomes from goat seminal plasma were isolated by differential centrifugation and serial ultracentrifugation. TEM observation showed that the collected vesicles displayed typical double membrane structures and round-shape morphology with diameters at approximately 100 nm (Fig. 4A). The diameters of vesicles were distributed in the range of 30–200 nm and peaked at 142 nm (Fig. 4B). The expressions of exosome specific marker protein Alix, HSP70 and CD63 were detected in the obtained spEXs by Western blot analysis (Fig. 4C), with the absence of Calnexin protein (as a negative control). In addition, all the proteins were expressed in the samples of goat spermatozoa, but they were barely found in the supernatant of seminal plasma after removing exosomes. These results validated the successful isolation of high-density exosomes from seminal plasma.

To further validate the interaction of exosomes with spermatozoa, the DiO-labeled spEXs (green fluorescence) were incubated with spermatozoa and detected at different time points (Fig. 4D). After 0.5 h, the green fluorescence signal was observed surrounding the spermatozoon tail, indicating the touching of spEXs towards the spermatozoa. After 1 h, a small number of exosomes with green fluorescence were found in spermatozoa mitochondria. With the increase in incubation time, more and more spermatozoon mitochondria exhibited green fluorescence, and the fluorescence intensity gradually enhanced. In addition, the external fluorescence signal at the surrounding gradually weakened until disappeared. These results indicated that spEXs could bind and interact with spermatozoon mitochondria under liquid storage.

### The spEXs improved motility and OXPHOS of goat spermatozoa during liquid storage

**Fig. 5:**
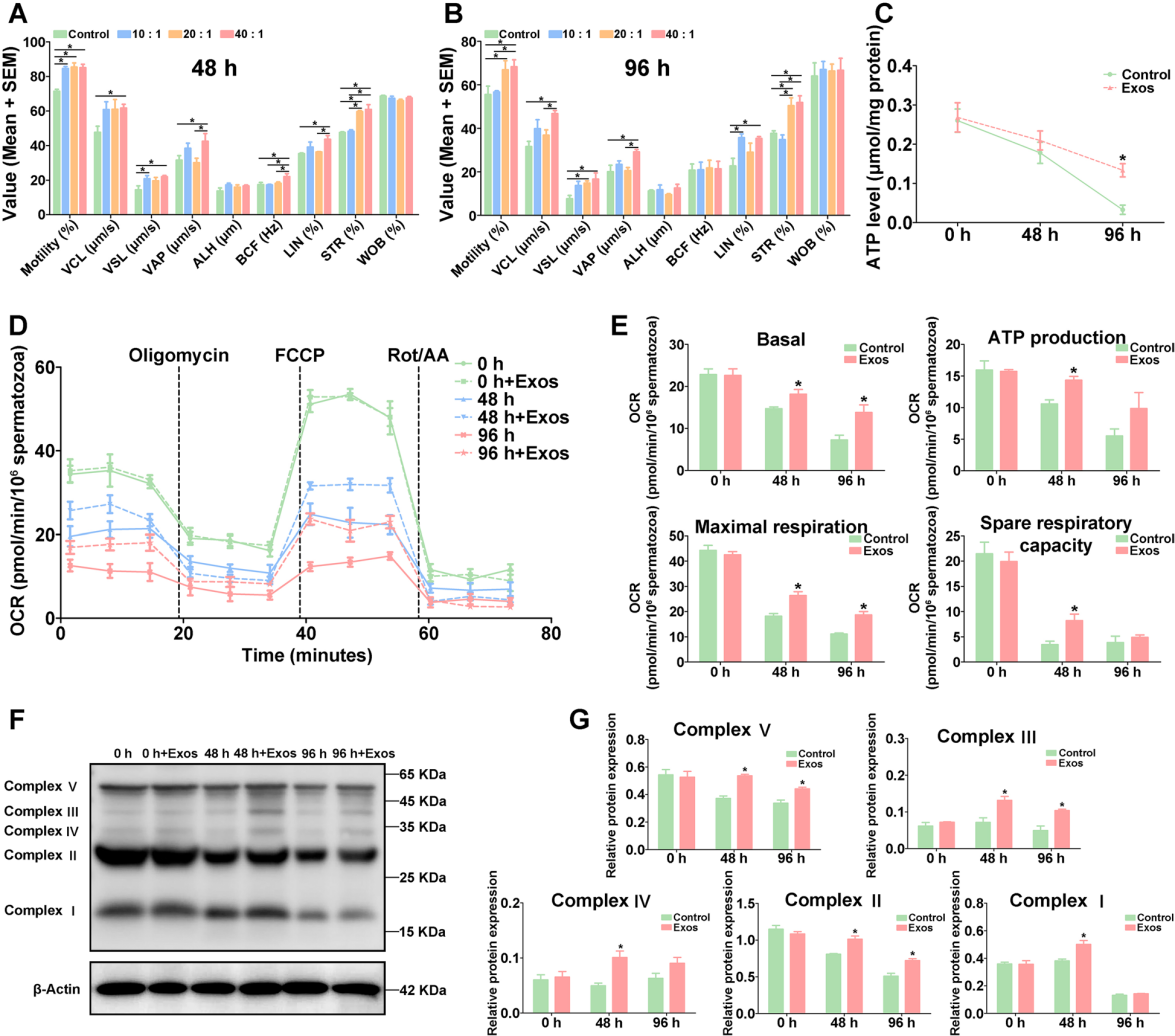
Effects of seminal plasma exosome application on motility parameters (**A**, **B**), ATP level (**C**), oxygen consumption rate (**D**, **E**), and oxidative phosphorylation complex protein expression (**F**, **G**) in goat spermatozoa under liquid storage. 10:1, 20:1, and 40:1 indicate the ratios of exosomes to spermatozoa. VCL, curvilinear velocity. VSL, straight-line velocity. VAP, average path velocity. ALH, amplitude of lateral head displacement. BCF, beat cross frequency. LIN, linearity. STR, straightness. WOB, wobble. OCR, oxygen consumption rate. *P < 0.05

To assess the effects of spEXs on liquid-stored goat spermatozoa, the motility of spermatozoa was evaluated after spEXs supplement with different concentrations. The results showed that the motility of liquid-stored spermatozoa in the presence of all concentrations of exosomes was significantly improved (*p* < 0.05) compared with that in the control group at 48 h (Fig. 5A). Meanwhile, the VSL of spermatozoa in EX-10 group (exosome to spermatozoa concentration ratio of 10:1) and the STR of spermatozoa in EX-20 group (exosome to spermatozoa concentration ratio of 20:1) was significantly increased (*p* < 0.05), and the VCL, VSL, VAP, BCF, LIN, and STR of spermatozoa in EX-40 group (exosome to spermatozoa concentration ratio of 40:1) were also significantly increased (*p* < 0.05) (Fig. 5A). Moreover, the VAP, BCF, LIN, and STR of spermatozoa showed significant increase in EX-40 group compared to that in EX-10 and EX-20 groups at 48 h (Fig. 5A). After 96 h, the LIN of spermatozoa was significantly elevated (*p* < 0.05) in EX-10 group, and a significant increase (*p* < 0.05) was detected in the motility, VSL, and STR of spermatozoa in EX-20 group, as well as in the motility, VCL, VSL, VAP, LIN, and STR of spermatozoa in EX-40 group (Fig. 5B). In addition, the motility, VCL, VAP, and STR of spermatozoa showed significant increase in EX-40 group compared to that in EX-10 and EX-20 groups at 96 h (Fig. 5B). In general, the application of spEXs obviously improved the motility of goat spermatozoa under liquid storage, especially in EX-40 group, and thus the addition of spEXs to spermatozoa at the ratio of 40:1 was used in the following assays.

Further analysis revealed that the ATP level of liquid-stored goat spermatozoa after incubating with spEXs was significantly higher (*p* < 0.05) at 96 h than that in control group (Fig. 5C), although the ATP content gradually decreased with the increase in liquid storage time. The results of OCR analysis showed that spEXs significantly elevated the mitochondrial respiratory function of spermatozoa and the indicators of OXPHOS (*p* < 0.05) (Fig. 5D, E), including basal respiratory, ATP production, maximal respiratory, and spare respiratory capacity. Moreover, the expression levels of mitochondrial OXPHOS complex proteins were significantly enhanced after incubating with spEXs at 48 h, and complex V, III, and II expressions were significantly increased at 96 h (Fig. 5E, G). These results suggested that the application of spEXs could promote the level of mitochondrial OXPHOS in liquid-stored goat spermatozoa.

### The spEXs improved mitochondrial function of goat spermatozoa during liquid storage

**Fig. 6:**
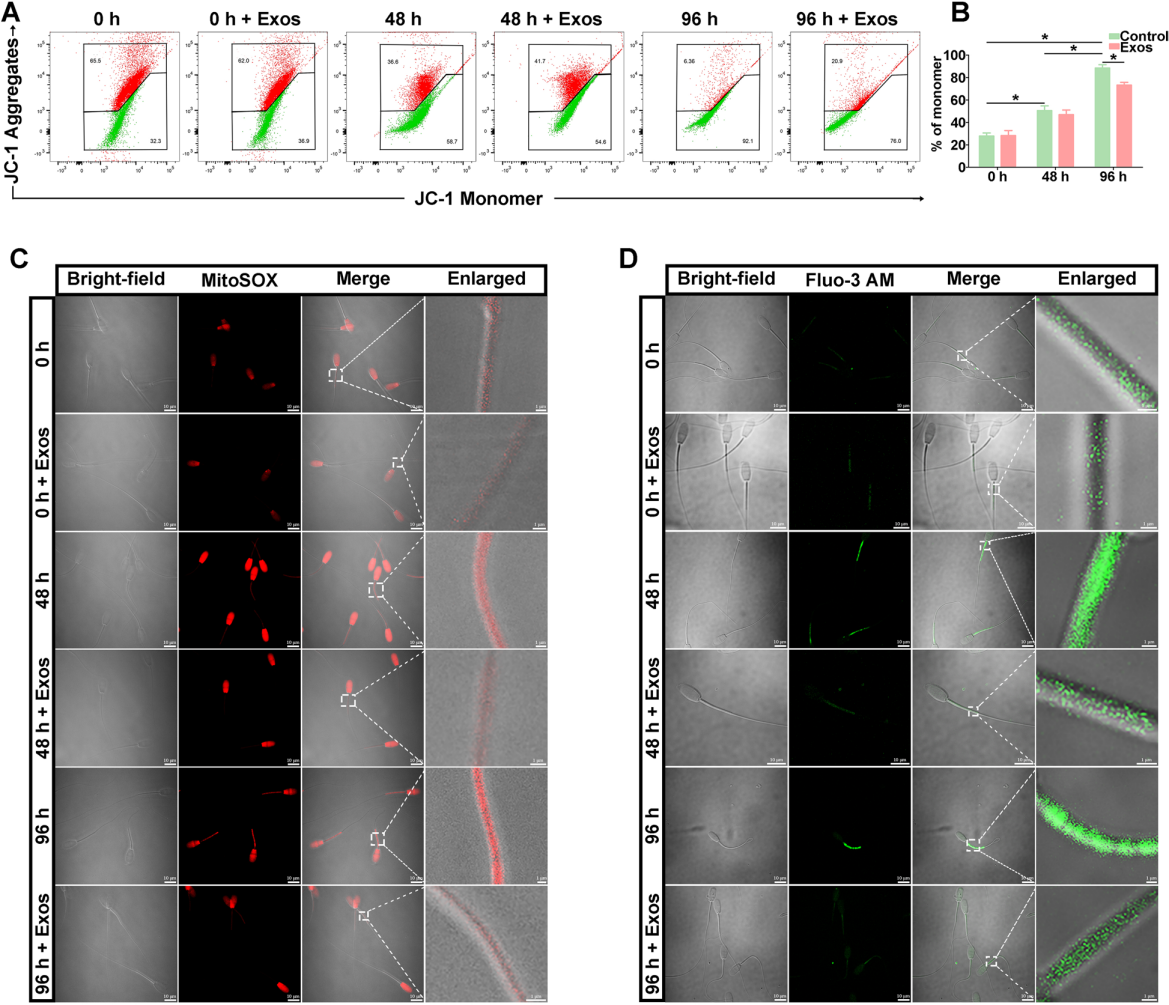
Effects of seminal plasma exosome application on mitochondrial membrane potential (MMP), the level of mitochondrial reactive oxygen species (ROS), and intracellular Ca^2+^ content in goat spermatozoa under liquid storage. **A**, **B** JC-1 staining displayed the changes in MMP. *P < 0.05. **C** MitoSOX Red staining showed the mitochondrial ROS content. **D** Intracellular Ca^2+^ content was showed using Fluo-3 AM staining

Mitochondrial dysfunction is a crucial determinant of the decline in the quality of liquid-stored spermatozoa (Boguenet et al. 2021; Durairajanayagam et al. 2021). To evaluate the effects of spEXs on mitochondrial function under liquid storage, the MMP and mitochondrial reactive oxygen species (mtROS) level were detected after incubating with spEXs. The results of JC-1 staining showed a significant decline (*p* < 0.05) in MMP of liquid-stored goat spermatozoa at 48 h and 96 h, which exhibited the decreased JC-1 aggregation (red) and increased JC-1 monomer (green) (Fig. 6A, B). After incubating with spEXs, a significant improve (*p* < 0.05) in MMP with the increased JC-1 aggregation (red) and decreased JC-1 monomer (green) was observed at 96 h (Fig. 6B), indicating that the addition of spEXs improved the MMP in spermatozoa. Furthermore, the mtROS content was measured using MitoSOX Red staining. The red fluorescence representing the mtROS level was gradually increased (*p* < 0.05) with the liquid storage time (Fig. 6C). As expected, a significant decrease in the levels of mtROS was observed in the spEXs-treated groups, suggesting that spEXs might attenuate mitochondrial oxidative damage under liquid storage.

In addition, the intracellular Ca^2+^ concentration of liquid-stored spermatozoa was detected to further determine the effects of spEXs on mitochondrial function. The green fluorescence intensity around spermatozoon tail was gradually enhanced with the liquid storage time, indicating the increased Ca^2+^ level in mitochondria (Fig. 6D). Remarkably, the spEXs-treated spermatozoa exhibited a significantly decrease in green fluorescence intensity at 48 h and 96 h compared to the control group, representing the reduced Ca^2+^ level. These results indicated that the addition of spEXs could alleviate liquid storage-induced mitochondrial impairment on goat spermatozoa.

### The spEXs may transfer TFAM protein to goat spermatozoa to maintain mtDNA under liquid storage

**Fig. 7:**
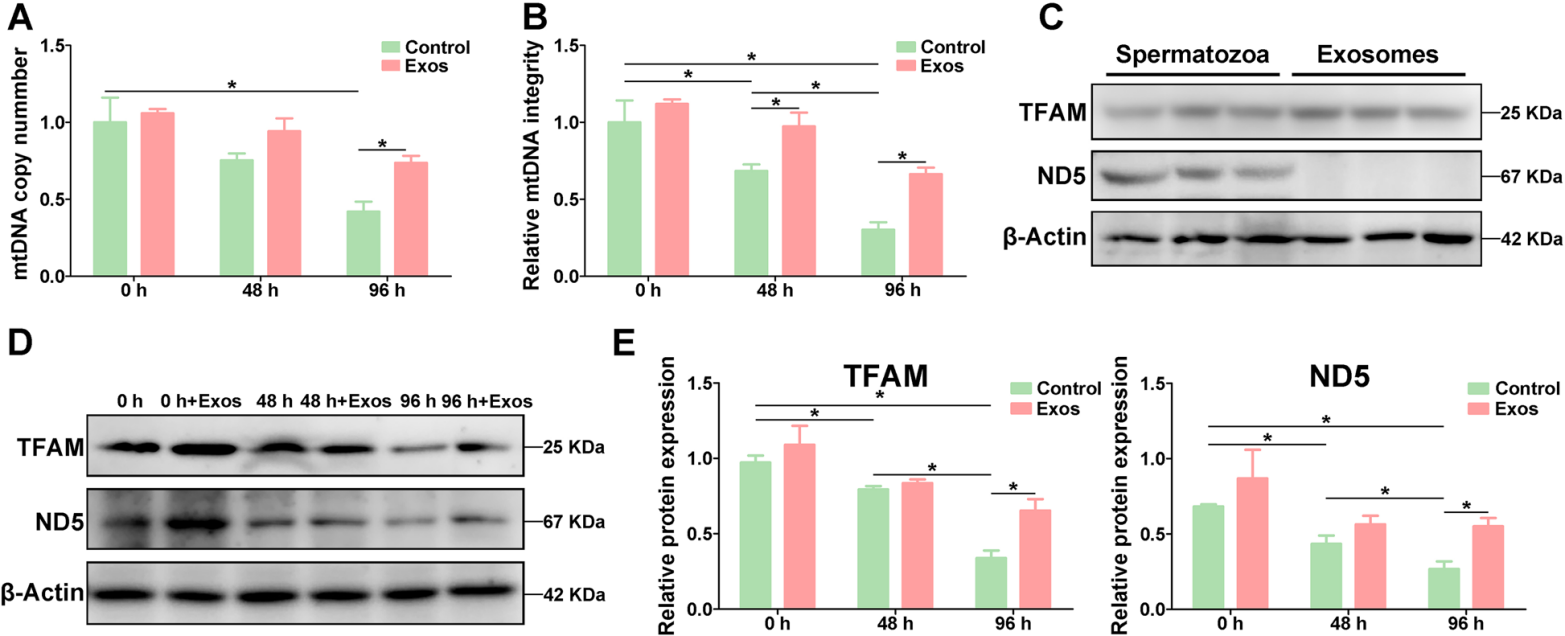
Improvement of the copy number (**A**) and integrity (**B**) of mitochondrial DNA and expression analysis of TFAM and ND5 proteins (**C**, **D**, **E**) in goat spermatozoa after incubating with seminal plasma exosomes under liquid storage. *P < 0.05

The copy number and integrity of mtDNA are closely associated with the mitochondrial function (Boguenet et al. 2021; Durairajanayagam et al. 2021). In this study, the mtDNA copy number was significantly decreased (*p* < 0.05) at 96 h under liquid storage (Fig. 7A), and the mtDNA integrity was also significantly decreased (*p* < 0.05) at 48 h and 96 h under liquid storage (Fig. 7B). Notably, after incubating with spEXs, a significant increase (*p* < 0.05) in the copy number was detected at 96 h compared to that in control group (Fig. 7A). Meanwhile, the integrity of mtDNA was also significantly increased (*p* < 0.05) in spEXs-treated group at 48 h and 96 h compared to that in control group (Fig. 7B). These results suggested that spEXs could reduce liquid storage-induced mtDNA damage.

The above proteomic analysis found that the mitochondrial OXPHOS related ND5 protein was significantly down-expressed in liquid-stored spermatozoa (Fig. 1C). Western blot analysis showed that the ND5 protein level was gradually decreased with the liquid storage time, but a significant increase (*p* < 0.05) in ND5 expression was observed in the spEXs-treated groups at 96 h by comparing to the corresponding control group (Fig. 7D, E), which was consistent with the estimation results of mtDNA integrity and copy number. However, ND5 protein expression was not detected in the isolated spEXs (Fig. 7C). Notably, the expression of TFAM protein was obviously observed in the samples of spEXs (Fig. 7C). Furthermore, although TFAM protein level was gradually decreased under liquid storage, the expression of TFAM was higher in the spEXs-treated spermatozoa than that in control group and exhibited significant increase at 96 h (*p* < 0.05) (Fig. 7D, E), inferring that the enhanced TFAM may derive from spEXs. Overall, these results indicated that the application of spEXs could maintain mtDNA by transferring TFAM protein to improve the mitochondrial function of goat spermatozoa under liquid storage.

## Discussion

The proper management and long-time preservation of semen are the vital determinants for the practicing efficiency and success of artificial insemination in goat breeding (Faigl et al. 2012; Zuidema et al. 2021). Liquid storage of goat spermatozoa at reduced temperature is considered as a desirable method for spermatozoon in vitro preservation (Zuidema et al. 2021; Höfner et al. 2020). However, liquid storage remains under many intrinsic and extrinsic threats causing serious damages on morphological structure and physiological function of spermatozoa (Liu et al. 2019; Yang et al. 2018; Falchi et al. 2018; Quan et al. 2016), which directly limits the widespread application of this technology. Considerable reports documented that the decline in the motility of goat spermatozoon is one of the mainly detrimental consequences of liquid storage (Falchi et al. 2018; Sadeghi et al. 2020; Liu et al. 2016), and our previous study also revealed the time-dependent decrease in goat spermatozoon motility during liquid storage (Liu et al. 2019). Thus, to understand the underlying mechanism of the maintenance of spermatozoon motility is beneficial for the favorable liquid storage of goat spermatozoa. The present study further found that the parameters of motility and velocity were decreased gradually in goat spermatozoa during liquid storage. Meanwhile, the reduced ATP content of goat spermatozoa under liquid storage was detected along with the decrease in spermatozoon motility, indicating the close relationship of energy supply and spermatozoon motility (Amaral 2022; Tourmente et al. 2015; Kong and Sokolova 2024).

The adequate ATP supply is known to be crucial for preserving high motility of spermatozoa that determines the efficient use of spermatozoa (Ferramosca and Zara 2014; Durairajanayagam et al. 2021). Numerous evidences have demonstrated two mainly metabolic pathways of ATP production in mammalian spermatozoa (Ferramosca and Zara 2014; Tourmente et al. 2015), and a wide range of variation in the primary mechanism of ATP production for spermatozoon is represented among different animal species (Amaral 2022; Rodriguez-Gil and Bonet 2016). The specific structure of spermatozoa containing abundant mitochondria in the mid-piece offers evidences that mitochondrial OXPHOS pathway yields more ATP and is historically regarded as the main source of ATP production for spermatozoa (Gualtieri et al. 2021; Tourmente et al. 2015; Ferramosca et al. 2008). However, the importance of glycolysis pathway as the preferred energy source for spermatozoon motility had been reported by the earlier researches in human and mice (Mukai et al. 2004; Nascimento et al. 2008), and both glycolysis and OXPHOS pathways were considered equally important in several animals (Setiawan et al. 2024; Bucci et al. 2022; Losano et al. 2017). In this study, the difference in the energy metabolism related pathways was analyzed and showed that OXPHOS pathway rather than glycolysis pathway was significant enrichment in goat spermatozoa after liquid storage. Similarly, no significant difference was found in ECAR levels in liquid-stored goat spermatozoa, but the OCR indicators and OXPHOS complex protein levels were obviously decreased after liquid storage. These observations were consistent with the previous studies (Kong and Sokolova 2024; Sun et al. 2023; Fang et al. 2020), indicating that the decrease in OXPHOS is apparently more important for the reduced motility of goat spermatozoon under liquid storage.

The prime function of spermatozoon mitochondria is the production of ATP through OXPHOS pathway for supporting motility and function (Boguenet et al. 2021; Yin and Shen 2022). Extensive researches indicated that the decline in the motility and ATP content of spermatozoa is closely correlated with mitochondrial dysfunction (Liu et al. 2019; Durairajanayagam et al. 2021). To better understand the changes in physiological and biological processes of semen under liquid storage, the technique of HPF/FS was used to fix the semen samples for TEM observation in the current study. The conventional chemical fixation methods usually need to remove the seminal plasma from semen to observe spermatozoon, however, HPF/FS is suitable for water-rich samples like the semen by simultaneously fixing all macromolecules and more realistically and comprehensively displays the ultrastructural changes in semen, including exosomes and spermatozoa. In this study, the morphological results by HPF/FS-TEM revealed that the decrease in mitochondrial energy production may be attributed to the observed mitochondrial structural damage, such as mitochondrial swelling and deformity. Notably, the subsequent observation showed that many spEXs were found to surround the spermatozoa, and several spEXs seemed to bind and fuse with the spermatozoon mitochondria during liquid storage, which indicated the possible interaction of spEXs and spermatozoon mitochondria. Numerous studies have confirmed the involvement of spEXs in the regulation of physiological processes of mammalian spermatozoa (Mahdavinezhad et al. 2022; Wang et al. 2022; Kowalczyk and Kordan 2024; Parra et al. 2024). In the present study, the high-density exosomes with typical round-shape morphology and size ranging from 30–200 nm were obtained from goat seminal plasma, which was accordant with the other reports (Mahdavinezhad et al. 2022; Rodriguez-Caro et al. 2019). Furthermore, the isolated spEXs were labeled by DiO marker and used to incubate with the liquid-stored spermatozoa. As expected, the green fluorescence representing spEXs was detected around the incubated spermatozoa, especially at spermatozoon mitochondria, and the fluorescence intensity gradually enhanced with the increase in incubation time. These findings further suggested the binding and fusing of spEXs with spermatozoon mitochondria, which may be significant in maintaining the motility of spermatozoa during liquid storage.

The seminal plasma is a complex fluid secretion that has been perceived as a transport medium and provides a nutrient-rich environment for spermatozoa during and after ejaculation (Parra et al. 2024; Rodriguez-Martinez et al. 2021). Considering the detrimental effects of the existence of enzymes in seminal plasma to spermatozoa, such as phospholipase A, yolk agglutinase, and glycoprotein-60, the absence or reduced presence of seminal plasma is generally applied in the common procedures of goat spermatozoon liquid storage (Cabrera et al. 2005; Zou et al. 2022). Nevertheless, the beneficial components in seminal plasma, especially exosomes, are noteworthy to elucidate the critical roles in the improvement of spermatozoon quality and motility during storage (Zou et al. 2022; Wang et al. 2024). Exosomes carrying active biomolecules are widely found in seminal plasma and are important mediators for intercellular communication through the transfer of genetic materials (Guo et al. 2023; He et al. 2018). The vital roles of spEXs have been extensively reported to improve spermatozoon functional properties. Studies on boar had revealed the effects of spEXs in regulating mitochondrial metabolism by interacting with the spermatozoa to control the parameters of spermatozoon motility, such as progressive motility, viability, and ATP production (Zou et al. 2022; Guo et al. 2019; Du et al. 2016). The report by Mahdavinezhad et al. (2022) also showed that the addition of spEXs to human spermatozoa increased all the parameters related to the structure and physiological functions of spermatozoa after cryopreservation. In this study, the effects of spEXs on liquid-stored goat spermatozoa were investigated, and the incubation of spEXs significantly improved the motility of spermatozoa and ATP content. Moreover, the OCR parameters and the mitochondrial respiratory function were significantly elevated in spEXs-incubated spermatozoa under liquid storage. The increase in the expression levels of mitochondrial OXPHOS complex proteins in spermatozoa was also found after incubating with spEXs. These results indicated that spEXs could promote the maintenance of motility and energy generation of liquid-stored goat spermatozoa through the enhanced mitochondrial OXPHOS, which was in line with previous reports (Mahdavinezhad et al. 2022; Guo et al. 2019; Wang et al. 2024).

Mitochondrial damage and oxidative stress induced by liquid storage or cryopreservation are mainly responsible for affecting spermatozoon motility (Boguenet et al. 2021; Durairajanayagam et al. 2021). Many studies are devoted to suppress the mitochondrial functional impairment to achieve the improvement of spermatozoon motility and the consequent successful fertilization (Moraes and Meyers 2018; Durairajanayagam et al. 2021). For instance, a study by Fang et al. (2020) reported that the addition of melatonin conferred protection on mitochondria from cryoinjury and improved OXPHOS and ATP synthesis of frozen-thawed ram spermatozoa. It has been demonstrated that the high concentration of ROS is associated with impaired motility and viability of spermatozoa, and the altered MMP as indication of mitochondrial damage and dysfunction has a deleterious effect on spermatozoon quality (Boguenet et al. 2021; Gualtieri et al. 2021). In this study, a significant decrease in the level of mtROS and the repaired MMP were found in spEXs-treated spermatozoa under liquid storage, suggesting that spEXs could eliminate excess mtROS and the mitochondrial damage to liquid-stored goat spermatozoa. Meanwhile, the Ca^2+^ level in spEXs-incubated spermatozoa was reduced, which revealed the crucial roles of spEXs in regulating the intracellular Ca^2+^ homeostasis by protecting mitochondria. Moreover, mitochondrial OXPHOS depends on the activity of multi-enzymatic complexes that are encoded by mtDNA, and the copy number and integrity of mtDNA has been reported to determine the mitochondrial function and the following spermatozoon motility (Boguenet et al. 2021; Durairajanayagam et al. 2021; Popova et al. 2022). Interestingly, the spEXs-incubated goat spermatozoa exhibited significant improvement in the copy number and integrity of mtDNA as expected, strongly supporting the positive roles of spEXs in alleviating the liquid storage-induced mitochondrial damage to goat spermatozoa.

More importantly, accumulating evidences have shown the roles of spEXs in maintaining spermatozoon function by allowing the transport of biochemicals such as mRNAs, miRNAs, and proteins (Wang et al. 2024; Du et al. 2016; Piehl et al. 2013). Mitochondrial TFAM plays critical roles in modulating cellular mitochondrial metabolism and biogenesis, such as mtDNA replication, transcription, and stability (Rantanen et al. 2001; Song et al. 2020). Previous studies had suggested that TFAM deficiency led to severe mtDNA depletion, mitochondria damage and aberrant OXPHOS (Gao et al. 2022; Huang et al. 2022). In this study, the expression of TFAM was increased in spEXs-treated spermatozoa than that in control group, which was consistent with the increase in mtDNA integrity, the repaired MMP, and the decrease in the mtROS and Ca^2+^ levels after spEXs treatment. Moreover, the presence of TFAM protein was detected in goat spEXs. These results suggest that the enhanced TFAM proteins found in spEXs-treated spermatozoa may derive from the added spEXs, and the increase in TFAM was valuable for mitochondrial improvement. Remarkably, although the increased ND5 protein expression was also detected in spEXs-treated spermatozoa, the absence of ND5 protein expression was found in the isolated spEXs, which indicated that the increased ND5 protein in spermatozoa was not transferred by spEXs. It is known that ND5 protein belongs to one of the subunits of ETC complexes and is encoded by mtDNA (Boguenet et al. 2021; Popova et al. 2022), and its expression depends on the integrity of mtDNA and is affected by mitochondrial damage (Durairajanayagam et al. 2021). The increase in ND5 level in spEXs-treated spermatozoa may be attributed to the improvement of mtDNA that was regulated by TFAM. Thus, the spEXs-induced improvement of mtDNA integrity and mitochondrial function may contribute to the increase in TFAM expression in spEXs-treated goat spermatozoa.

## Conclusion

In this study, the exosomes from goat seminal plasma were used to validate the potential roles in regulating the motility and mitochondrial function of goat spermatozoa under liquid storage. Ultrastructural observation suggested the binding and fusing of exosomes with spermatozoon mitochondria by HPF/FS-TEM. Furthermore, the addition of goat spEXs exhibited the improvements of motility, OXPHOS level, and mitochondrial functional parameters in liquid-stored spermatozoa. Moreover, a significant increase in TFAM was also found in exosome-treated spermatozoa. In general, these results of this study indicate that the goat spEXs could maintain spermatozoon motility and facilitate improving OXPHOS and mitochondrial function, perhaps through delivering TFAM protein to spermatozoa under liquid storage.

## Materials and methods

### Ethical approval

All procedures involving animals were approved by the Institutional Animal Care and Use Committee of Northwest A&F University, China (Approval No. DY2023033), with every effort made to minimize animal suffering.

### Goat semen collection and processing

Semen was collected from 6 healthy purebred Guanzhong dairy goats (*Capra hircus*) aged about 3 years using an artificial vagina, with 48 h interval between each collection. The semen with normal odor and color, concentrations > 3 × 10^9^ spermatozoa/mL and > 90% motility were retained and pooled. The pooled semen was diluted to 3 × 10^8^ spermatozoa/mL with a tris-citrate-glucose extender (Tris 3.63 g/100 mL, fructose 0.50 g/100 mL, citric acid 1.99 g/100 mL, egg yolk 10 mL/100 mL, penicillin 5,000 IU/100 mL, and streptomycin 0.1 g/100 mL) at 37 ℃. After dilution, the semen samples were wrapped with 10 layers of gauze and kept in a 4 °C refrigerator.

### Analysis of spermatozoon motility and ATP content

The motility of spermatozoa was assessed using a computer assisted sperm analysis (CASA) system CEROS II (Hamilton Thorne Biosciences, MA, USA). For analysis, at least 200 spermatozoa and five random fields were selected for each sample, and the following parameters were evaluated: motile spermatozoa (Motility, %), VCL (μm/s), VSL (μm/s), VAP (μm/s), ALH (µm), BCF (Hz), LIN (%), STR (%), and WOB (%). Motile spermatozoa were defined as VCL > 5 μm/s and VAP > 5 μm/s.

The ATP content of spermatozoa was measured with an ATP Assay Kit (S0026, Beyotime, Shanghai, China) according to the previous study (Liu et al. 2023). Briefly, the semen samples were washed and then dissociated by RIPA lysis buffer (R0278, Sigma-Aldrich, MO, USA). After that, the spermatozoon supernatant was mixed with luciferase reagent in 96-well plates. A microplate reader (Bio-Rad, CA, USA) was used to measure luminescence. The protein concentrations in the spermatozoa lysate were determined using BCA protein assay kit (23,225, Thermo Scientific, MA, USA) and were used for normalization.

### Gene set enrichment analysis (GSEA)

The entire proteomic expression data of goat spermatozoa under liquid storage at 0 h, 48 h, and 96 h were taken from our previous study (Liu et al. 2019). GSEA (https://www.gsea-msigdb.org/gsea/index.jsp) of the KEGG data set was performed using the GSEA tool (Broad Institute, MA, USA) as described previously (Subramanian et al. 2005). A *p*-value of < 0.01 and false discovery rate (FDR) with a *q*-value of < 0.1 were considered as statistically significant.

### Assessment of ECAR and OCR

The ECAR and OCR of spermatozoa were assessed using the Seahorse XF96 Flux Analyzer (Agilent, CA, USA). The glycolytic stress assay (103,020–100, Agilent, CA, USA) and mitochondrial stress assay (103,015–100, Agilent, CA, USA) were used for ECAR and OCR detection, respectively. In brief, spermatozoa were seeded in the 96-well XF Seahorse incubation plate at a density of 1 × 10^6^ spermatozoa per well as the protocol indicated. Following that, spermatozoa were treated with glucose (10 mM), oligomycin (1 μM), and 2-DG (50 mM) at specific time points according to the manufacturer’s guidelines for the measurement of ECAR. Similarly, oligomycin (1 μM), FCCP (1 μM), and rotenone/antimycin A (both 1 μM) were added for the detection of OCR value. The values obtained in each measurement for triplicate wells were averaged and were displayed as the ECAR (mpH/min/10^6^ spermatozoa) and OCR (pmol O_2_/min/10^6^ spermatozoa).

### Western blot analysis

Total protein was extracted using RIPA lysis buffer (R0278, Sigma-Aldrich, MO, USA) supplemented with a protease inhibitor cocktail (P8340, Sigma-Aldrich, MO, USA). Equal amounts of protein were separated by SDS-PAGE, transferred onto polyvinylidene fluoride (PVDF) membranes, and incubated overnight at 4 ℃ with primary antibodies followed by HRP-conjugated secondary antibody (1:1000, ab6802, Abcam). Reacting bands were visualized using ECL reagent (32,209, Thermo Scientific, MA, USA), and the intensity of the bands was analyzed using Quantity One V 4.62 software (Bio-Rad, CA, USA). Primary antibodies employed in this study included anti-β-actin (1:1000, ab8227, Abcam), anti-total OXPHOS cocktail (1:1000, ab317270, Abcam), anti-Alix (1:1000, 92,880, Cell Signaling Technology), anti-Hsp70 (1:1000, ab181606, Abcam), anti-CD63 (1:1000, ab315108, Abcam), anti-Calnexin (1:5000, ab92573, Abcam), anti-ND5 (1:1000, ab230509, Abcam), and anti-TFAM (1:1000, ab307302, Abcam).

### High-pressure freeze fixation and freeze substitution

The semen samples were high-pressure frozen and freeze substituted (HPF/FS) following a previously described protocol with slight modifications (Jadav et al. 2023). In brief, semen samples were loaded onto 200 µm deep copper gold-plated membrane carriers (Leica, Vienna, Austria), coated with hexadecane and immediately frozen in an EM ICE High Pressure Freezer (Leica, Vienna, Austria) after reaching 2100 bar pressure. After that, the frozen samples were transferred into a freeze-substitution chamber (EM AFS2, Leica, Vienna, Austria) under liquid nitrogen, where they were freeze substituted for 60 h at −90 °C in 2% OsO_4_ and 0.1% uranyl acetate in acetone. Subsequently, the temperature was gradually increased (5 °C/h) to −20 °C, maintained at −20 °C for 16 h, and then the chamber temperature raised to 20 °C (2.5 °C/h). Samples were washed in acetone, stepwise embedded in increasing concentrations of Epon 812 in acetone and polymerized at 60 °C for 72 h.

### Transmission electron microscopy

Ultrathin Sects. (70 nm) of embedded semen samples were prepared with an ultramicrotome (EM UC7, Leica, Vienna, Austria), counterstained with 2% uranyl acetate and Reynold’s lead citrate. Sections were imaged with a TEM (Tecnai G2 Spirit; FEI, OR, USA) at 80 kV.

For identifying the ultrastructure of exosomes, exosome suspension was dropped onto carbon-coated electron microscopy grids and allowed it to dry at room temperature for 20 min. Then, exosomes were stained with 2% uranyl acetate for 10 min and imaged by TEM (Tecnai G2 Spirit; FEI, OR, USA).

### Isolation and characterization of spEXs

Exosomes in seminal plasma were isolated as previously described with some modifications (Wang et al. 2021). Briefly, fresh semen was diluted with PBS (1:1), centrifuged at 1000 g for 15 min, 3000 g for 15 min and 10,000 g for 25 min at 4 °C, to eliminate cells, cellular debris, and larger vesicles. The resulting supernatant filtered through 0.22-μm sterile filter (Millipore, MA, USA) and ultracentrifuged at 120,000 g for 90 min at 4 °C. The pellets were resuspended in PBS, pooled, and stored at −80 °C for further investigations. A Nanosight NS300 system (NanoSight, Malvern, UK) was applied to analyze the distribution of vesicle diameters from the exosomes. The concentrations of added exosomes were n-fold that of spermatozoa. According to the experimental requirements, the exosomes were divided into tenfold (3 × 10^9^ particles/mL), 20-fold (6 × 10^9^ particles/mL), and 40-fold (12 × 10^9^ particles/mL), respectively, and were used to incubate with spermatozoa.

### Validation of spermatozoa interacting with exosomes

Exosomes were labeled with the fluorescent lipophilic dyes DiO (V22886, Invitrogen, CA, USA) at 37 °C for 30 min. The free dye was washed away by ultracentrifugation at 120,000* g* for 2 h to obtain DiO-labeled exosomes. Then, the DiO-labeled exosomes were seeded on spermatozoa and co-cultured at 4 °C. The uptake of exosomes by spermatozoa was visualized by the confocal laser scanning microscope (LSM980, Carl Zeiss, Jena, Germany).

### Measurement of MMP, mtROS and intracellular Ca^2+^

The MMP of spermatozoa was assessed using the JC-1 kit (T3168, Invitrogen, CA, USA). The density of spermatozoa was adjusted to 1 × 10^6^ spermatozoa/mL. Next, spermatozoa were assembled with the JC-1 fluorescent probe in darkness at 37 °C for 30 min. After washed with PBS, MMP was measured by flow cytometry (FACSAria, BD Biosciences, CA, USA).

The mtROS levels in spermatozoa were assessed using MitoSox Red (M36008, Invitrogen, CA, USA) according to the manufacturer’s instructions. Briefly, spermatozoa were incubated with MitoSox Red (2 μM) for 30 min at 37 °C. After washing with PBS, mtROS was visualised by the confocal laser scanning microscope (LSM980, Carl Zeiss, Jena, Germany).

Intracellular Ca^2+^ of spermatozoa was analyzed using Fluo-3 AM staining (F1241, Invitrogen, CA, USA). Briefly, the washed spermatozoa (1 × 10^6^ spermatozoa/mL) were labeled with 5 μM Fluo-3 AM at 37 °C for 30 min. After washing, the fluorescence intensity was measured with a confocal laser scanning microscope (LSM980, Carl Zeiss, Jena, Germany).

### Analysis of copy number and integrity of mtDNA

The relative mtDNA copy number of spermatozoa was determined using a fluorescence-based quantitative real-time PCR (RT-qPCR) method as previously described (Zhang et al. 2024). Briefly, total DNA was extracted from goat spermatozoa using a DNA extraction kit (9770A, Takara, Dalian, China) according to manufacturer’s instructions. The mtDNA copy number analysis was based on the expression ratio of mitochondrial gene (*ND5*) to a nuclear gene (*β-actin*). The sequences of primers were as follows: ND5 forward: 5′-TCATCCTCGTCACCGCAAAT-3′ and reverse: 5′-GTGTTTGCGTCTGTTCGTCC-3′; β-actin forward: 5′-CTCACTGCTTCCTCCTCTCTCC-3′ and reverse: 5′-CTAGAAGCATTTGCGGTGGAC-3′. RT-qPCR was carried out in triplicate on a CFX96 real-time PCR system (Bio-Rad, CA, USA). The relative expression values are calculated by 2^−ΔΔCT^.

For mtDNA integrity, the long-range PCR was used as described previously with a few modifications (Zhang et al. 2020). The primer pair 5′-CCTAGATGAGTGTACCAACTCCA-3′ and 5′-AGTGGACGGGATACGCATGTT-3′ was used to amplify the mtDNA genome. The long-range PCR was performed with 200 ng of spermatozoon DNA using TaKaRa LA (RR02MA, Takara, Dalian, China) in a 50 μL reaction system as follows: 95 °C for 2 min, 30 cycles of 95 °C for 15 s, 62 °C for 1 min, 68 °C for 15 min, and a final extension at 72 °C for 10 min. The products were then checked on a 1% agarose gel stained with ethidium bromide. The relative mtDNA integrity was calculated as a ratio of the intensity of the band versus mtDNA copy number.

### Statistical analysis

All data were represented as the mean ± SEM of at least three independent experiments. The statistical analysis was conducted using SPSS version 20 (IBM, NY, USA). Student’s *t*-test was used to determine the statistical significance of two groups, and the One-way ANOVA was used to assess multiple comparison. *P* values < 0.05 was considered statistically significant.

## Supplementary Information

Additional file 1: Fig. S1 Gene set enrichment analysis of oxidative phosphorylation (A, B) and glycolysis/gluconeogenesis (C, D, E) pathways in goat spermatozoa at 48 h and 96 h after liquid storage.

## Data Availability

All data and materials included in this study are available upon request by contact with the corresponding author.

## Abbreviations

ATP: Adenosine triphosphate
OXPHOS: Oxidative phosphorylation
ETC: Electron transport chain
mtDNA: Mitochondrial DNA
TFAM: Mitochondrial transcription factor A
spEXs: Seminal plasma exosomes
GSEA: Gene set enrichment analysis
ECAR: Extracellular acidification rate
OCR: Oxygen consumption rate
HPF/FS: High pressure freezing/freeze-substitution
TEM: Transmission electron microscope
MMP: Mitochondrial membrane potential
mtROS: Mitochondrial reactive oxygen species
RT-qPCR: Quantitative real-time PCR
