# Supplementary figures and images for: Seminal plasma exosomes improve the motility and mitochondrial function of goat spermatozoa during liquid storage by regulating oxidative phosphorylation
Source: Stress Biol. 2025 Oct 28;5(1):65. doi: 10.1007/s44154-025-00253-6 (PMC12569307; doi:10.1007/s44154-025-00253-6)

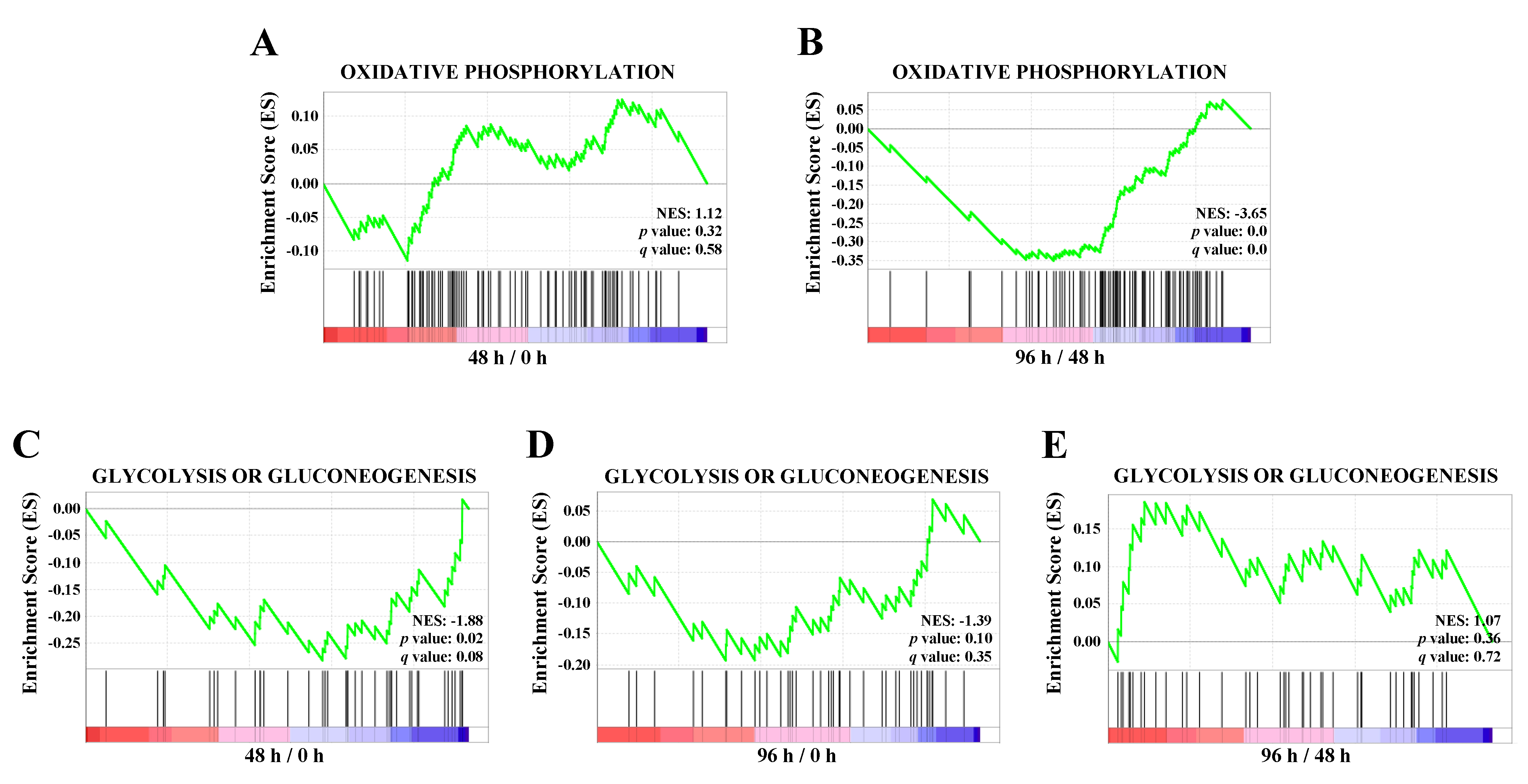

Supplement: Supplementary file 1 — Additional file 1: Fig. S1 Gene set enrichment analysis of oxidative phosphorylation (A, B) and glycolysis/gluconeogenesis (C, D, E) pathways in goat spermatozoa at 48 h and 96 h after liquid storage. [file 44154_2025_253_MOESM1_ESM.jpg]
